# Revealing the Second and the Third Causes of AgNPs Property to Restore the Bacterial Susceptibility to Antibiotics

**DOI:** 10.3390/ijms24097854

**Published:** 2023-04-26

**Authors:** Nina Bogdanchikova, Maria Maklakova, Luis Jesús Villarreal-Gómez, Ekaterina Nefedova, Nikolay N. Shkil, Evgenii Plotnikov, Alexey Pestryakov

**Affiliations:** 1 Centro de Nanociencias y Nanotecnología, Universidad Nacional Autónoma de México, Ensenada 22800, BC, Mexico; 2 Facultad de Pedagogía e Innovación Educativa, Universidad Autónoma de Baja California, Av. Monclova Esq con Calle Río Mocorito S/n, Ex-Ejido Coahuila, Mexicali 21360, BC, Mexico; 3 Facultad de Ciencias de la Ingeniería y Tecnología, Universidad Autónoma de Baja California, Blvd. Universitario 1000, Unidad Valle de Las Palmas, Tijuana 22260, BC, Mexico; 4 Siberian Federal Scientific Centre of Agro-BioTechnologies of the Russian Academy of Sciences, 630501 Novosibirsk, Russia; 5 Tomsk National Research Medical Center of the Russian Academy of Sciences, Mental Health Research Institute, 634014 Tomsk, Russia; 6 Research School of Chemistry and Applied Biomedical Sciences, Tomsk Polytechnic University, 634050 Tomsk, Russia

**Keywords:** antibiotics resistance, increase of bacterial susceptibility to antibiotics, silver nanoparticles, anti-lysozyme activity, adhesion, bovine mastitis

## Abstract

The increase in bacterial resistance to antibiotics is a global problem for public health. In our previous works, it was shown that the application of AgNPs in cow mastitis treatment increased *S. aureus* and *S*. *dysgalactiae* susceptibility to 31 antibiotics due to a decrease in the bacterial efflux effect. The aim of the present work was to shed light on whether the change in adhesive and anti-lysozyme activities caused by AgNPs also contribute to the restoration of bacterial susceptibility to antibiotics. In vivo sampling was performed before and after cow mastitis treatments with antibiotics or AgNPs. The isolates were identified, and the adhesive and anti-lysozyme activities were assessed. These data were compared with the results obtained for in vitro pre-treatment of reference bacteria with AgNPs or antibiotics. The present study revealed that bacterial treatments in vitro and in vivo with AgNPs: (1) decrease the bacterial ability to adhere to cells to start an infection and (2) decrease bacterial anti-lysozyme activity, thereby enhancing the activity of lysozyme, a natural “antibiotic” present in living organisms. The obtained data contribute to the perspective of the future application of AgNPs for recovering the activity of antibiotics rapidly disappearing from the market.

## 1. Introduction

The frequent use of antibiotics in the agricultural sector, in particular in livestock, leads to growth in the resistance of bacteria that cause high animal mortality [1]. The remnants of antibiotics used for the treatment of cows, pigs, and poultry when meat, eggs, milk, and milk products are consumed by humans also contribute to the growth of bacterial resistance [2]. The World Health Organization has recognized antibiotic resistance as one of the prevailing modern problems since May 2015 [3].

As an alternative to antibiotics, metallic nanoparticles have been gaining great interest [4]. In previous works of our group, it was shown that the application of AgNPs in cow mastitis treatment increased the susceptibility to antibiotics [5,6]. It was shown that this phenomenon at least partially can be explained by the decrease in the capacity of bacteria to efflux (to flow out) the antibiotics [5,6]. However, it is known that the efflux effect is one of a wide variety of methods microorganisms use to survive and reproduce. Other virulence factors include adherence to cells and mammalian lysozyme opposition [7,8]. Colonization is a crucial phenomenon for bacterial pathogenicity [9] because it is necessary for the pathogen to stick to the host cell to start an infection. Colonization and subsequent internalization facilitate the delivery of toxins and virulence factors to the host cell and help the bacteria maintain their position and resist host immunity [9]. Lysozyme is a natural “antibiotic” secreted by cells of the immune system of humans and animals such as macrophages and neutrophils, and capable of destroying the peptidoglycan of the bacterial cell wall. In addition to the antimicrobial action, lysozyme is also involved in the regulation of inflammation. Hence, the destruction of the lysozyme by bacteria can lead both to an increase in the virulence of bacteria and to secondary effects in the host body [10].

Mastitis is the most frequent and costly cow disease that provokes a negative impact on the economic profit in the industry due to milk losses and therapy investments [11,12]. More than 130 bacterial strains of pathogens were found in bovine mastitis probes [12], where the *Streptococcus genus* [12], *Staphylococcus genus* [13], and *Escherichia coli* [14,15] are the most common bacterial families that induce bovine mastitis. Antibiotics proposed for mastitis treatment have resulted as effective in decreasing bacterial infection and curing bovine mastitis. However, antibiotics used for mastitis prevention and cure can be accumulated in milk, which gives rise to food safety concerns [4]. Thus, the effect of AgNPs pre-treatments on the resistance of bacteria common for bovine mastitis is important from fundamental and practical points of view.

The aim of the present work was to shed light on the question of whether the change of adhesive and anti-lysozyme activities caused by AgNPs treatment contribute to returning bacterial susceptibility to antibiotics. For these experiments, Argovit-C^TM^ AgNPs was chosen because, for this specific formulation, the effect of an increase in bacterial susceptibility to antibiotics was revealed [6].

## 2. Results

### 2.1. Number of Isolates with Different Bacteria (In Vivo)

**Figure 1:**
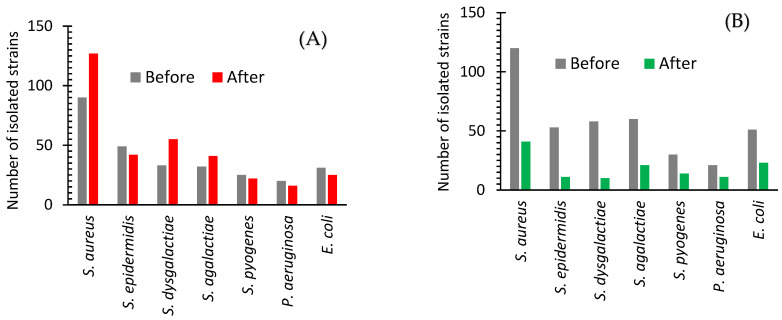
Isolated strains of bacteria from cows with subclinical mastitis before and after treatment with an antibiotic drug (**A**) or AgNPs (**B**).

**Figure 2:**
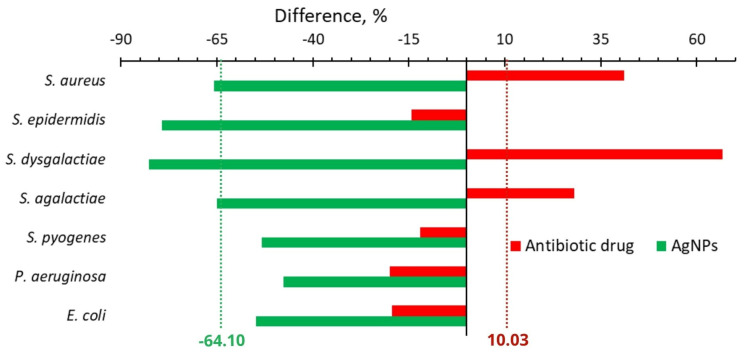
Difference in the number of isolated bacteria from cows with mastitis after treatment with an antibiotic drug (red bars) or AgNPs (green bars) and before the treatment.

Figure 1A shows the difference between the number of isolated strains of seven bacteria from 150 cows with a subclinical form of mastitis before and after the treatments with Lactobay (an antibiotic drug commonly used in farming). The frequency of the excretion of such bacteria as *S. epidermidis*, *S. pyogenes*, and *P. aeruginosa* slightly decreased and such bacteria as *S. aureus*, *S. dysgalactiae*, and *S. agalactiae* increased (Figure 1A). The number of isolates of the main causative agent of cow mastitis, *S. aureus*, after antibiotic drug treatment increased significantly (Figure 1A and Figure 2).

Figure 1B shows a significant decrease in the frequency of detection of all major causative agents of cow mastitis after the use of AgNPs. The number of isolates of these bacteria after AgNPs treatment decreased as compared with one before the treatment in the range from 53 to 83% (Figure 1B and Figure 2). The most significant changes were found for *S. dysgalactiae*, where the number of isolates changed from 58 to 10 (83% decrease); for S. *epidermidis*, where it dropped from 53 to 11 (79% decrease); and for *S. aureus*, where it diminished from 120 to 41 (68% decrease). The average difference in the number of isolated bacteria before and after the treatment with the antibiotic drug and AgNPs was +10.03 and −64.10%, respectively (Figure 2).

### 2.2. Anti-Adhesion Activity

#### 2.2.1. In Vitro

**Figure 3:**
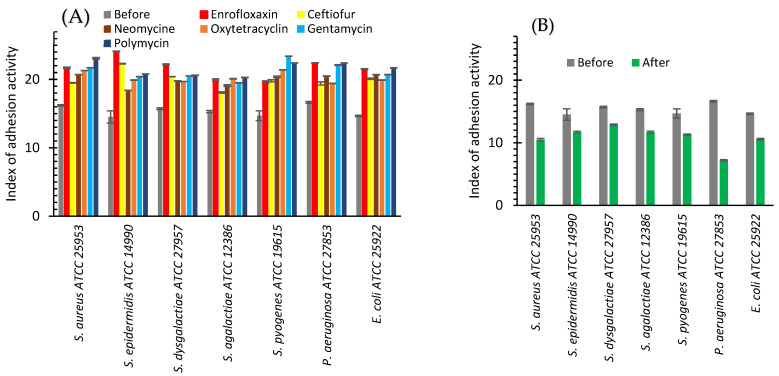
Index of adhesive activity of bacteria before (grey bars) and after cultivating with: antibiotics (enrofloxaxin—red bars, ceftiofur—yellow bars, neomycin—brown bars, oxytetracycline—orange bars, gentamicin—green bars, polymycin—blue bars) (**A**) or AgNPs (green bars) (**B**).

Exposure to bacteriolytic (beta-lactams, fluoroquinolones) and bacteriostatic (macrolides, tetracycline, aminoglycosides) antibiotics during the cultivation of the reference (ATCC) bacterial strains with an initially high adhesion index (greater than 4) in a non-lethal dosage led to the increase in the average adhesion index for all studied bacteria (Figure 3A). Exposure of the reference bacterial strains to antibiotics with non-lethal dosage led to an increase in the adhesion index for all studied bacteria from 18.1 to 24.1 (Figure 3A). For *S. aureus*, the adhesion index after cultivation with the antibiotics increases from 16.21 to an interval of 19.5–23.1 depending on the antibiotic with maximum growth for polymyxin. For *S. dysgalactiae* the observed adhesion index increased from 15.73 to 19.7–22.2.

The reverse effect was observed after cultivating bacteria with AgNPs (Figure 3B). Before AgNPs exposure, the average adhesion index varied from 14.52 to 16.65%. After AgNPs exposure, it dropped to the interval from 7.2 to 12.9% for different bacteria. The maximum drop was observed for *P. aeruginosa*, where the adhesion index decreased from 16.65 to 7.2%, and for *S. aureus*, where it was diminished from 16.21 to 10.5% (Figure 3B).

**Figure 4:**
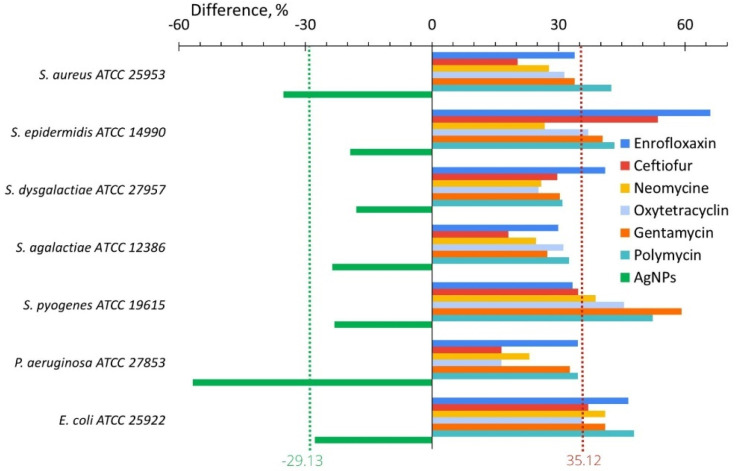
Difference of the bacterial adhesive activity between its values after and before in vitro treatment with AgNPs (green bars) or antibiotics (another color bars).

Figure 4 illustrates the difference between the values of the adhesion index after and before in vitro bacterial exposure to antibiotics and AgNPs. This difference was a minimum (16.52) for *P. aeruginosa* after the treatment with oxytetracycline and a maximum (65.98) for *S. epidermidis* after enrofloxacin. The effect of AgNPs was reversed and the adhesion index difference varied from −17.99% for *S. dysgalactiae* to −56.76% for *P. aeruginosa.* The average adhesion index difference for studied bacteria was 35.12% for antibiotics and −29.13% for AgNPs (Figure 4).

#### 2.2.2. In Vivo

**Figure 5:**
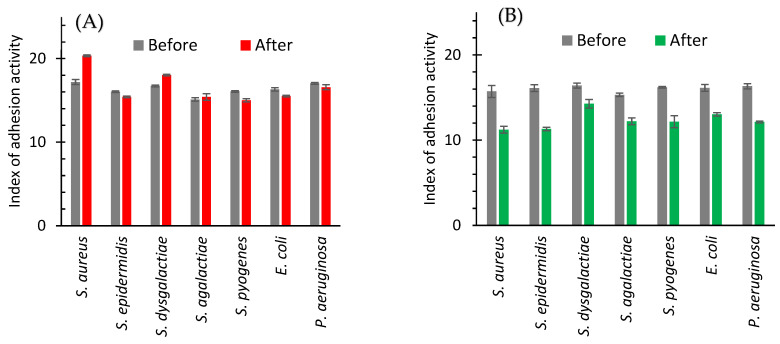
Index of adhesive activity of bacteria isolated from milk of cows with subclinical mastitis before and after treatment with antibiotic drug (**A**) or AgNPs (**B**).

In in vivo study, the adhesive ability of bacterial strains isolated from the milk after treatment with antibiotics changed only slightly (Figure 5A). The adhesion index increased in the case of *S. aureus* (from 17.19 to 20.33%) and *S. dysgalactiae* (from 16.72 to 18.03%) and practically remains unchanged or insignificantly decreases in the case of the other bacteria (Figure 5A).

**Figure 6:**
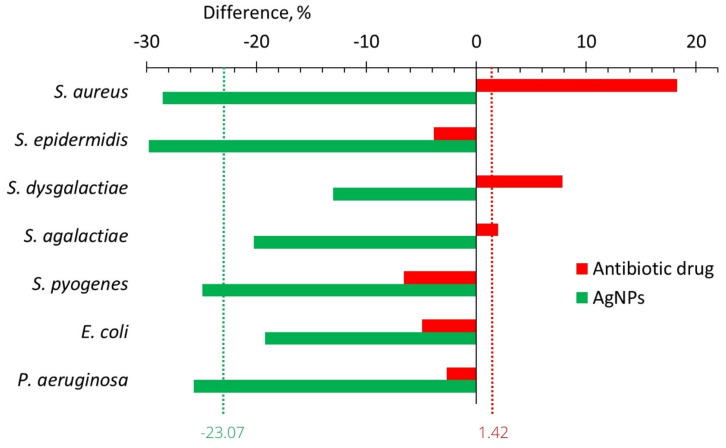
The change in the adhesion index of isolated strains of bacteria between values after and before the treatments with the antibiotic drug (red bars) and AgNPs (green bars).

After cow treatment with AgNPs the clearly defined decrease in the index of adhesive activity for all bacteria isolated from milk was registered (Figure 5B). The smallest effect (from 16.41 to 14.27%) was observed for *S. dysgalactiae*. A highest decrease was noticed for *S. epidermidis* (Figure 5B and Figure 6).

Figure 6 summarizes the effect of cow treatments with an antibiotic drug and AgNPs on the adhesive activity of the isolated strains of microorganisms. The average adhesion index after the use of the antibiotic drug increased by 1.42%, while after the use of AgNPs, it decreased by 23.07% (Figure 6).

### 2.3. Anti-Lysozyme Activity

#### 2.3.1. In Vitro

**Figure 7:**
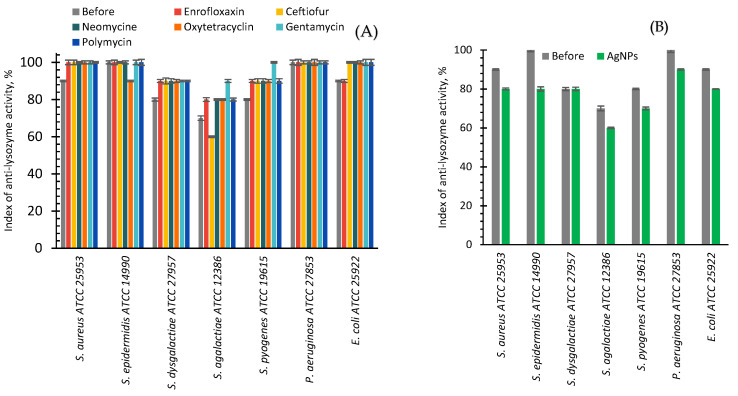
Index of anti-lysozyme activity of bacteria before (grey bars) and after cultivating with antibiotics (various colors bars, **A**) or AgNPs (green bars, **B**).

The anti-lysozyme activity of the bacteria studied in this work before treatments was maximal (100%) in the case of *S. epidermidis* and *P. aeruginosa* (Figure 7A). The anti-lysozyme activity of *S. aureus* and *E. coli* raised from 90 to 100% after their cultivation with antibiotics. For the majority of antibiotics in the case of *S. dysgalactiae*, *S. agalactiae*, and *S. pyogenes* after the antibiotic treatments, a 10 percent increase of anti-lysozyme activity was observed from 80 to 90%, from 70 to 80%, and from 80 to 90%, respectively. However, after gentamycin treatment, a 20% increase was observed for *S. agalactiae* and *S. pyogenes*. Only for *S. agalactiae* and *S. epidermidis* after the use of ceftiofur and oxytetracycline, respectively, a 10% decrease was registered (Figure 7A).

After in vitro cultivation of seven strains of reference bacteria with AgNPs, a 10% decrease in anti-lysozyme activity was found for six bacteria, and the absence of changes for *S. dysgalactiae* (Figure 7B).

**Figure 8:**
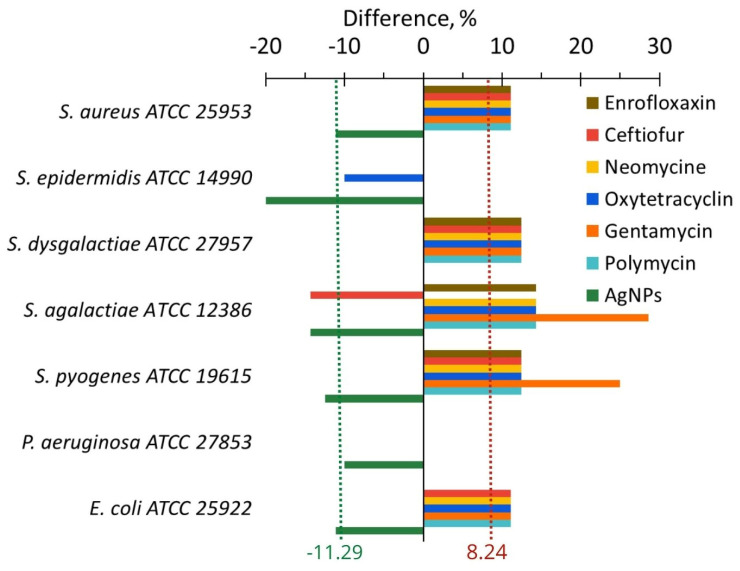
The difference in the anti-lysozyme activity of bacteria between the values after and before cultivating with AgNPs (green bars) and antibiotics (other color bars).

The difference in in vitro influence of antibiotics or AgNPs on anti-lysozyme activity is clearly visible in Figure 8. AgNPs reduced it by an average of 11.29%, while antibiotics increased it by an average of 8.24%. However, some exceptions to the general trend were observed. For example, oxytetracycline and ceftiofur decreased the anti-lysozyme activity of *S. epidermidis* and *S. agalactiae*. In addition, some antibiotics and AgNPs for *S. dysgalactiae* did not provoke any change in the anti-lysozyme activity.

#### 2.3.2. In Vivo

**Figure 9:**
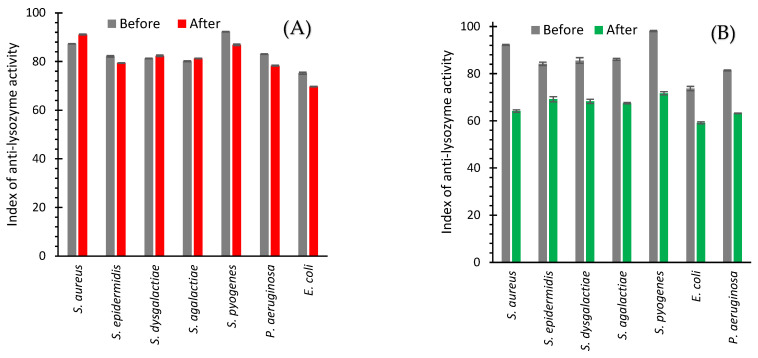
Anti-lysozyme activity of isolated strains of bacteria from cows with subclinical mastitis before and after treatment with an antibiotic drug (**A**) and AgNPs (**B**).

In the study of isolated bacterial strains from the milk of cows with subclinical mastitis confirmed by the California test, it was found that the index of anti-lysozyme activity of all studied strains was in the interval of 75.2–81.3% (Figure 9A,B). Antibiotic drug treatment had a relatively insignificant effect on this parameter (Figure 9A). The increase in the index of anti-lysozyme activity was observed for such bacterial isolates as *S. aureus* (from 87.3 to 91.1%), *S. dysgalactiae* (from 81.3 to 82.4%), and *S. agalactiae* (from 80.1 to 81.3%). The rest of the bacterial isolates showed a slight decrease (Figure 9A).

The use of AgNPs for the treatment of cows led to the fast normalization of the level of bacterial milk contamination, which was confirmed by the California test. Complete cow recovery after AgNPs treatment was observed after 2.9 days, while after treatment with an antibiotic drug, it was observed only after 7.1 days. It showed that AgNPs treatment was 2.5 times faster than antibiotic drug treatment.

**Figure 10:**
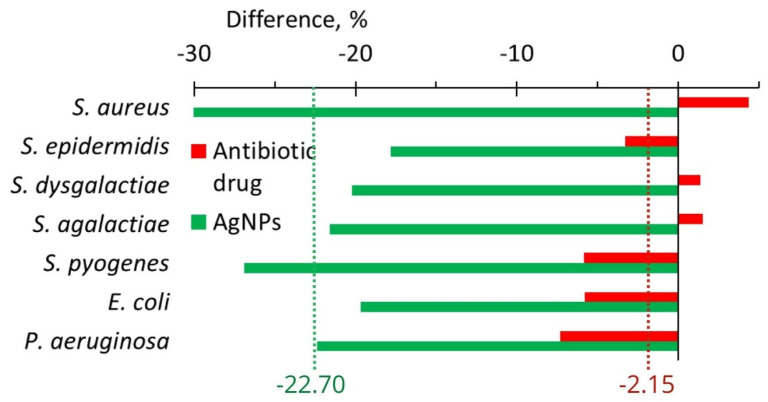
The difference in anti-lysozyme activity of isolates between the values after and before treatments with an antibiotic drug (red bars) and AgNPs (green bars).

AgNPs treatment led to a decrease in the anti-lysozyme activity of isolates of all seven bacteria from the cow milk (Figure 9B). A maximum drop from 92.2 to 64.2% was revealed for *S. aureus* (Figure 9B and Figure 10). The use of an antibiotic drug practically did not change the anti-lysozyme activity of isolated bacterial strains because the average value showed a decrease of only 2.15% (Figure 10). However, AgNPs treatment led to a reduction of anti-lysozyme activity by 22.70% (Figure 10).

## 3. Discussion

### 3.1. Isolated Bacteria Strains

In our study, seven bacteria were detected causing mastitis such as *S. epidermitis*, *S. pyogenes*, *P. aeruginosa*, *S. aureus*, *S. dysgalactiae*, *S. agalactiae*, and *E. coli* (Figure 1). The Streptococcus family (*S. agalactiae*, *S. dysgalactiae*, and *S. uberis*) was the most common species found in mastitis bovine [12]. These results do not contradict the results of the present work, where high populations of *S. aureus*, *S. agalactiae*, and *S. dysgalactiae* were revealed (Figure 1). In our work, *S. aureus* was the most abundant strain isolated from cow milk (Figure 1). *S. aureus* is the predominant pathogen responsible for mastitis in farm cows being community-acquired or a nosocomial infection agent [13] contaminating raw milk, formatting biofilms and toxins, etc. [16]. In our study, the opportunistic pathogen *E. coli* also was found. These bacteria cause inflammation of the udder in dairy cows, resulting in reduced milk production, changes in milk composition and quality, and even cow death [14].

The conventional treatment for mastitis and streptococcal infection consists of the use of β-lactam antibiotics such as enrofloxacin, erythromycin, and tetracycline [17]. In our case, Lactobay antibiotic drug containing two β-lactam antibiotics (ampicillin and cloxacillin) was applied. Obtained results showed that after in vivo antibiotic drug treatment, the number of isolates of seven bacteria populations on average slightly increased (+10%, Figure 1A). This observation shows the effect of growth in bacterial resistance to antibiotics. The effect of the increase of bacterial resistance to antibiotics was observed to a much greater extent in some works; for example, Reding-Roman et al. (2019) [18] found that resistance of *E. coli*, that was exposed to 8 rounds of doxycycline treatments for 4 days, continued to increase with each treatment. The population of mutated *E. coli* after these treatments increased three times compared with one pre-treatment [18].

In contrast, after AgNPs treatment, the average number of isolates for seven bacteria decreased considerably (−64%, Figure 1B and Figure 2), indicating that the application of AgNPs did not lead to growth in bacterial resistance. These results illustrate that the integral benefit from in vivo application of AgNPs instead of an antibiotic drug is 74.1% (Table 1).

The 64% decrease in the number of isolated strains after AgNPs treatment (Figure 2) can be explained by well-known antibacterial properties of AgNPs, the ability of AgNPs to inhibit the bacterial efflux pump activity [5,6], and the capacity of AgNPs to decrease the bacterial adhesive ability and anti-lysozyme activity, as shown in the present work (Table 1).

In this work, we found that the total benefit from AgNPs use compared with antibiotic use was 74% in terms of the decrease in the number of isolates (Table 1). It indicates a strong antibacterial effect of AgNPs, which, together with a 2.5-times reduction of the duration of treatment (Table 1), showed the preference for AgNPs use over antibiotic drug use for cow subclinical mastitis treatment.

### 3.2. Adhesion Activity

In in vitro study, for all seven bacteria, which are frequent in cow mastitis, after the application of AgNPs, the average adhesive activity decreased by 29.1%; while, after the application of seven antibiotics, it increased by 35.2% (Figure 4, Table 1). After in vivo treatment with AgNPs, the adhesive activity of all seven bacteria decreased on average by 23.1%, while after treatment with the antibiotic drug, it was practically unchanged (increased by 1.45%, Figure 6, Table 1). For both in vitro and in vivo experiments, we observed the same tendency. AgNPs application leads to a decrease in bacterial adhesive activity, while antibiotics lead to its increase or its changelessness. The total benefit in adhesive activity from AgNPs application compared with antibiotics application is more significant for experiments in vitro (64.2%, Table 1) than in vivo (24.5%, Table 1).

For pathogens, it is essential to adhere to the host cell to begin the infection. A decrease in adhesion can lead to a decrease in infection level. This approach is known as anti-adhesion therapy, which uses various techniques such as the application of analogs of receptor and adhesion, dietary components, antibiotics sublethal concentrations, and adhesion-based vaccines [9]. The majority of compounds, which were studied for application in anti-adhesion therapy, are complex organic compounds found in living organisms or plants [9]. In Table 2, some examples of these compounds (plant extracts, chitosan, salvianolic acid B, complex of multivalent adhesion molecules with polystyrene microbeads) with a quantitative change of adhesive activity are presented.

Bacteria adhesive activity usually decreases or changes slightly after the application of subinhibitory concentrations of antibiotics varying with antibiotic type, its concentration, and bacteria strains [25,26]. For ATCC 25922, MTCC 729, and U 105 *E. coli* strains in the case of ciprofloxacin, ceftazidime, gentamicin, ampicillin, and cotrimoxazole, a 20–1000% drop in adhesive activity was observed [27]. However, there are some works, which describe the rise of bacteria adhesive activity under the action of antibiotics. For example, an increase in adhesive capacity was observed for *E. coli* under quinolones [28,29], and *S. aureus* under rifampin action [30]

Table 2 includes two articles dedicated to the study of the influence of nanoparticles (AgNPs, TiO_2_NPs, and WO_3_NPs) on bacteria adhesive activity. As can be seen from Table 2, the percentage of adhesive activity in these cases decreased by 74–99%. In our case, the decrease was between 23 and 29%. For adequate comparison of the literature and our data, measurements should be conducted under the same experimental conditions (using the same adhesive activity method, the same bacteria, the bacteria concentrations, nanoparticle concentrations incubation time, pH, etc.). It would be interesting for further experiments to do such a comparison of Argovit AgNPs with other nanoparticle formulations to estimate the potential of Argovit AgNPs in anti-adhesion therapy.

### 3.3. Anti-Lysozyme Activity

After in vitro application of AgNPs, the anti-lysozyme activity for seven bacteria important for cow mastitis decreased on average by ~11.3%; while, after the application of six antibiotics, in contrast, it increased by 8.2% (Figure 8, Table 1). In vivo AgNPs treatment led to a 22.7% decrease in the anti-lysozyme activity of studied bacteria, while antibiotic drug treatment led only to a 2.2% average decrease (Figure 10, Table 1). The cumulative benefit from AgNPs application compared to antibiotic use was very close for in vitro and in vivo experiments (19.5 and 20.5%, respectively) (Table 1).

There are very few articles devoted to the study of the anti-lysozyme activity of different compounds in the literature. However, we managed to find two works dedicated to in vivo mastitis treatments. Shabunin et al. (2020) [31] evaluated the effect of Biferon-B (the drug, exhibiting antiviral and immunostimulant activity, which presents a mixture of bovine recombinant a- and y-interferons with species specificity) on the prevention of mastitis in cows by intramuscular injection of Biferon- B. Biferon-B was able to increase lysozyme activity by 4.3–13.7%. This increase was less than in our case (20.5%). The methodology reported led to preventive effect in 25.0–75.0% of the cows [31]. Unfortunately, the drug showed an alteration effect on immunological cells such as neutrophils (~59–65%), monocytes (~6–57%), and lymphocytes (~5–12%) [31]. Gao et al. (2017) [13] showed that the baicalin bioactive flavonoid binds to lysozyme increasing its activity against *S. aureus* isolated from mice with mastitis. In this study, *S. aureus* counts dropped in proportion to the baicalin concentration. At 80 µg/mL of baicalin, the lysozyme activity increased twice, while in our case it increased only by 23%. In our case, the AgNPs application in vivo corresponded to the optimized cow mastitis treatment protocol that limited the variation of AgNPs concentration. In the future, variation in AgNPs concentration should be conducted for a deeper understanding of the capacity of AgNPs to decrease bacterial anti-lysozyme activity.

### 3.4. Problem of Bacteria Resistance to Antibiotics

The effects obtained in the present work for the application of antibiotics for subclinical mastitis cow treatment are well-known. Antibiotics are the first choice in mastitis treatment in cows, but their exaggerated use leads to the presence of antibiotic residues in human and animal food, promoting microbial resistance to antibiotics, which causes a negative impact on public health. All this generates many restrictions on uncontrolled antibiotic therapy in the dairy sector worldwide [32]. Bovine mastitis is an illness with high prevalence in the world; besides, it is one of the most important bovine pathologies, and the fight against this disease is the most expensive one in the dairy industry [11]. The review by El-Sayed A. and Kamel M. (2021) [32] exhorts the research community to develop novel therapeutic approaches to replace the use of antibiotics in mastitis control. These efforts can succeed due to nanotechnology, stem cell assays, molecular biological tools, and genomics. This review [32] discusses recent concepts to control mastitis such as the breeding of mastitis-resistant dairy cows, the development of novel diagnostic and therapeutic tools, the application of communication technology as an educational and epidemiological tool, the application of modern mastitis vaccines, cow drying protocols [33], teat disinfection, housing, and nutrition [32].

The present study is the extension of our earlier published translational studies [5,6], where AgNPs use was considered as a prospective approach to solving one of the most important problems in medicine consisting of the rapidly growing bacterial resistance to drugs. In these works [5,6], it was shown that cure of cow mastitis with Argovit^TM^ AgNPs formulation raises the susceptibility of *S. aureus* and *S. dysgalactiae* to thirty-one antibiotics, and vice versa, cure with antibiotic medicines Lactobay and Spectromast (first-line drugs for mastitis treatment) diminishes their susceptibility [5,6]. Antibiotic-containing drug treatments led to a 23–25% susceptibility loss to thirty-one antibiotics for these two bacteria. Vice versa, after the application of AgNPs, the susceptibilities grew by 11–13%. Moreover, AgNPs utilization accelerated cow mastitis healing by 33–40% compared with antibiotic drugs [5,6]. Important observations made in these works, regarding changes in the percentage of isolates with the efflux effect after cow treatment, shed light on the action of AgNPs as agents with the property to restore susceptibility to antibiotics. The percentage of bacteria isolates capable to efflux antibiotics after antibiotic drug treatment increased by 8–18%; while after AgNPs treatment, it decreased by 15–19%. This loss of the ability of bacteria to efflux (eliminate) antibiotics after AgNPs treatment was the first revealed cause of AgNPs’s property of restoring the susceptibility of bacteria to antibiotics.

The results of the present work revealed two more causes for AgNPs’s property to restore bacterial susceptibility to antibiotics. It was shown that the use of AgNPs decreased bacterial adhesive activity by 23–29% in in vitro and in vivo experiments, and the application of six antibiotics increase it by 35% in vitro and was practically unchanged (+2%) in in vivo experiments. The same tendencies were observed for bacteria anti-lysozyme activity. After AgNPs use, anti-lysozyme activity decreased by 11–23%; however, after antibiotic applications, it raised by 8% (in vitro) and was practically unchanged (−2%) in in vivo experiments. These results showed that AgNPs: (1) decreases the bacterial ability to steak to cells necessary for following cell infection and (2) decreases the bacterial anti-lysozyme activity. These are two effects increasing the capacity of AgNPs to restore bacterial susceptibility to antibiotics. Hence, the results of our two previous works [5,6] and the present work revealed that bacteria treatment in vitro and in vivo with AgNPs decreases the bacterial ability to develop resistance to drugs due to (1) a decrease of bacterial capacity to get rid of antibiotics, (2) a decrease in their ability to adhere to cells to start an infection, and (3) a decrease in bacteria anti-lysozyme activity, thereby increasing the activity of lysozyme, a natural “antibiotic” present in the living organisms.

The main purpose of a series of works including two previous [5,6] ones and the present one is the translation (converting) of results obtained in a fundamental study into knowledge being in the service of people. As far as we know, this series of our three publications is the pioneering work dedicated to in vivo fieldwork with 300–400 farm cows, which revealed the capacity of AgNPs to restore the bacteria susceptibility to antibiotics and shed light on this phenomenon. The results obtained in this work contribute to the perspective of the future application of AgNPs for recovering the activity of antibiotics disappearing from the market due to fast-growing bacteria drug resistance. Further work to expand the study of the effect of restoring bacterial susceptibility to antibiotics after the use of different AgNPs formulations is required.

## 4. Materials and Methods

### 4.1. Experimental Design

#### 4.1.1. In Vivo

The present work was carried out on 300 breeding farm cows with subclinical mastitis. The experimental protocol was approved by the Ethical Committee of the Federal State Budgetary Institution of the Siberian Federal Scientific Center for Agrobiotechnologies of the Russian Academy of Sciences (decision No. 00017 from 10 February 2017). The diagnosis was made based on clinical symptoms, followed by confirmation by a biochemical California test [34]. Full recovery was also established on the basis of a biochemical California test, which was conducted every day during treatment. Samples of the secretion of the mammary gland of cows (milk) from both groups were analyzed before and after their intracisternal treatment with Argovit-C^TM^ AgNPs or Lactobay^TM^. Samples taken before processing can be considered as reference one (control).

#### 4.1.2. In Vitro

The experimental design of in vitro experiments is described below in the Adhesive activity and Anti-lysozyme activity sections.

### 4.2. Sampling

**Figure 11:**
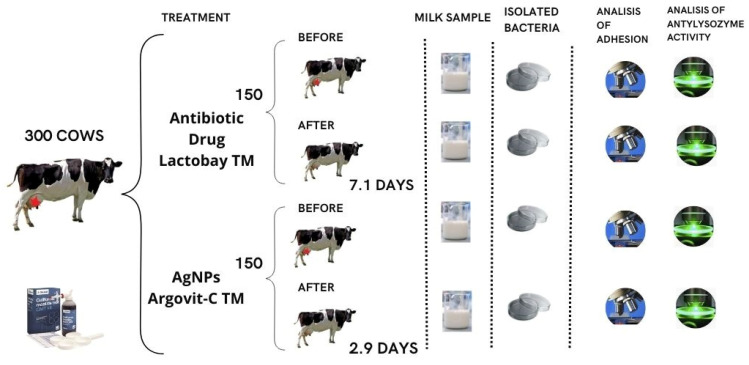
The scheme of treatment with the antibiotic drug and AgNPs for cows with subclinical mastitis.

Milk samples were taken from cows with mastitis before and after treatment with AgNPs or an antibiotic drug under the conditions of livestock farms in the Novosibirsk region from 2018 to 2019 (Figure 11). For sampling, the udder nipples were wiped with a cotton swab moistened with 70° ethyl alcohol. Milk in a volume of 10 mL was collected in clean test tubes avoiding contact with the nipple with the edge of the test tubes. The test tube with milk was closed with a cotton gauze stopper and the sample name and inventory number of the cow were recorded on the label of the test tube. The samples were stored at a temperature of 8–10 °C before the start of the tests. Within 3–4 h, samples were delivered for examination.

### 4.3. Treatment Formulations

#### 4.3.1. In Vitro

Enrofloxacin 50 (Hebei Yuanzheng Pharmaceutical, Co., Ltd., Shijiazhuang, China) is an antibacterial drug containing the active substance enrofloxacin 50 mg, and the excipients: sodium bisulfite—0.25 mg, potassium hydroxide—9 mg, ethylenediaminetetraacetic acid—0.2 mL, distilled water—up to 1 mL. Ceftiofur (JSC “BelVitunifarm”, Dolzha, Republic of Belarus) is an antibacterial drug of the third generation of the cephalosporin series; as an active ingredient, it contains 0.05 g of ceftiofur hydrochloride, the excipients: butylhydroxytoluene, benzyl alcohol, aluminum monostearate, fractionated sunflower oil. Neomycin sulfate (LLC NPP “Agrofarm”, Voronezh, Russia) is an antibacterial drug of the first generation of the aminoglycoside series. The content of neomycin as an active substance is 500 mg. Oxytetracycline hydrochloride (CJSC NPP “Agrofarm”, Voronezh region, Voronezh, Russia) is an antibacterial drug. The content of oxytetracycline hydrochloride as the active substance is not less than 860 µg/mg (in terms of dry matter). Gentamicin (JSC “Dalkhimpharm”, Moscow, Russia) is the antimicrobial drug of the aminoglycoside series with gentamicin as an active ingredient with a concentration of 40 mg/mL. Sodium disulfate (sodium metabisulfite), disodium edetate (disodium salt of ethylenediaminetetraacetic acid), and water for injection are the excipients. Polymyxin B (Jodas Expoim, Pvt. Ltd. (Hyderabad, Telangana, India) is an antimicrobial drug with 25 mg of polymyxin B sulfate. For all antibiotics in in vitro experiments, 100 µL of 10% solutions was used.

#### 4.3.2. In Vivo

Argovit-C^TM^ AgNPs formulation, produced by the Vector-Vita Research and Production Center, Novosibirsk, Russia as a veterinary drug, was provided by Dr. Vasily Burmistrov. It is used for therapeutic and prophylactic purposes for gastrointestinal diseases of calves. Argovit-C^TM^ AgNPs is a stable aqueous suspension of silver nanoparticles with a concentration of 200 mg/mL (20% by weight). The concentration of metallic silver is 12 mg/mL (1.2 wt.%), and the size of silver particles is in the range of 5–20 nm with an average diameter of 15 nm. AgNPs are stabilized with polyvinylpyrrolidone and collagen hydrolysate with a total concentration of 18.8 wt.%. The remaining 80% by weight corresponds to distilled water. In this work, the Argovit-C^TM^ formulation of AgNPs was studied. TEM data (JEM 2010 electron microscope, JEOL, Tokyo, Japan) showed that the metal silver particles are spherical with diameters varying in the range from 1 to 55 nm and with an average diameter of 14.95 ± 10.1 nm. In the UV-Visible spectrum (Cary 60 UV-Vis spectrophotometer, Agilent Technologies, Santa Clara, CA, USA), a peak typical of the plasma resonance of silver nanoparticles at 454 nm is observed. The hydrodynamic diameter has a bimodal distribution with maxima at 44 and 164 nm and the zeta potential is 9.6 ± 0.6 mV (Zetasizer Nanoseries Instrument, Nano-ZS, Malvern Instruments, Malvern, UK). A more detailed description of the physicochemical characterization of the Argovit-C^TM^ formulation of AgNPs is given in our previous work [35].

In in vitro experiments, original AgNPs was diluted 10 times, and 0.1 mL of the diluted solution was applied. For in vivo experiments, the Argovit-C^TM^ initial formulation was diluted 10 times and then it was administered to cows by intracisternal administration at a dose of 10 mL once a day for 3 days until complete recovery, confirmed by a biochemical California test.

Lactobay^TM^ antibiotic drug (“Norbrook Laboratories Limited”, Newry, Northern Ireland, UK) is antibacterial, which is the first-line drug for cow mastitis. It contains as active ingredients ampicillin (75 mg) and cloxacillin (200 mg) in the form of sodium salts, and it contains as excipients liquid paraffin (3.291 g) and white soft paraffin (up to 5.0 g). The antibiotic drug was administered to cows by intracisternal administration at a dose of 5 g twice per day with an interval of 12 h until recovery.

### 4.4. Isolation and Identification of Bacterial Isolates

#### 4.4.1. In Vivo

The bacteria *S. aureus*, *S. epidermidis*, *S. dysgalactiae*, *S. agalactiae*, *E. coli*, *P. aeruginosa*, and *S. pyogenes* isolated from the secret before and after treatment of cows with AgNPs or antibiotic drug were studied. Isolation of *Streptococcus* spp. and *Staphylococcus* spp. was carried out using a selective additive Condalab 6016 Staph-Strepto Supplement (Lab Unlimited Carl Stuart Group, Dublin, Ireland). Endo medium (FBUN SRC PMB Obolensk, Moscow, Russia) was used to isolate *E. coli.* The identification of microbiota isolated from animals was carried out by taking into account the cultural, morphological, and biochemical properties of bacteria according to generally accepted methods [36,37].

#### 4.4.2. In Vitro

In in vitro experiments, the following strains were used: *M. luteus* ATCC 15307, *S. aureus* ATCC 25953, *S. epidermidis* ATCC 14990, *S. dysgalactiae* ATCC 27957, *S. agalactiae* ATCC 12386, *S. pyogenes* ATCC 19615, *P. aeruginosa* ATCC 27853, *E. coli* ATCC 25922. The medium BTN-broth (nutrient broth) (Biotechnovatsiya LLC, Elektrogorsk, Russia) was used for the cultivation of microorganisms.

### 4.5. Adhesive Activity

The adhesive activity was evaluated according to the standard method of V.I. Brilis [38]. For this, the erythrocytes of healthy cows were washed three times with 0.1 M phosphate buffer solution by centrifugation. In this way, the suspension of erythrocytes with a concentration of 1.5 × 10^8^ cells/mL (McFarland 0.5 turbidity standard) was prepared. One inoculating loop of a broth culture of each strain and one drop of erythrocyte suspension were suspended on a clean fat-free slide. After 30 min of incubation at 37 °C in a wet chamber, the slides were dried and fixed with heat, then they were stained with aqueous fuchsin. The adhesive activity of each experimental culture was evaluated by immersion microscopy, counting the number of bacterial cells attached to 5 erythrocytes, and calculating the average adhesion index as an average. Microorganisms with average adhesion index 1.01–2.0, 2.01–4.0, and more than 4.0 were considered as low adhesive, medium adhesive, and highly adhesive, respectively. Densitometer Den-1B, Biosan (Ratsupites iela 7 k-2, Riga, Latvia) was used to determine the turbidity of the bacterial suspension. The experiment was carried out in a five-fold repetition.

### 4.6. Anti-Lysozyme Activity

The anti-lysozyme activity was evaluated by the standard method of O.V. Bukharin [39] using lysozyme (a lyophilized white powder) from Bryntsalov CJSC, Elektrogorsk, Russia. A solution of lysozyme was prepared at a concentration of 20 μg/mL in a phosphate buffer (KH_2_PO_4_—7.72 g, Na_2_HPO_4_·2H_2_O—1.78 g, distilled water—up to 1000 mL; pH 6.0–6.2).

The microbial mass of the studied cultures was seeded with a standard inoculating loop in 3 mL of liquid nutrient medium and cultured in a thermostat at a temperature of 37 °C for 24 h. The supernatant was separated from bacterial cells by centrifugation for 15 min at 3000 rpm. The grown bacterial cells of the test strain were killed with chloroform for 60 min, washed off, filtered through a coarse-pored filter, washed twice with phosphate buffer with trilon B and once with phosphate buffer (pH = 6.2).

The supernatant of the microorganism cultures (0.9 mL) was mixed with 0.1 mL of the prepared lysozyme solution and then was incubated for 60 min at 37 °C. Then, 0.5 mL of this mixture of supernatant and lysozyme was added to 2 mL of a suspension of the *M. luteus* ATCC 15,307 test culture (cultivated for 24 h), and the optical density of the resulting mixture was measured after 30 s in a Stat fax 2100 spectrophotometer (Awareness Technology, Inc., Palm City, FL, USA) at 492 nm against phosphate buffer. The experiment was carried out in a five-fold repetition.

### 4.7. Statistical Analyses

The results were statistically processed by parametric and nonparametric analysis methods. Statistical analysis was carried out using the program STATISTICA 13.3 (StatSoft Inc., Tulsa, OK, USA). ANOVA was performed with a 99.95% confidence interval.

## 5. Conclusions

In the present work, the influence of the application of six antibiotics from one side and AgNPs from the other side on the adhesive and anti-lysozyme activities of seven bacteria (frequent in cow mastitis) were studied in vitro and in vivo. The obtained results revealed two new properties of the Argovit AgNPs formulation consisting of decreasing bacteria adhesive and anti-lysozyme activities. After in vitro and in vivo application of AgNPs, adhesive activities decreased by 29 and 23%, respectively, and anti-lysozyme activities decreased by 11 and 23%, respectively. These properties facilitate the restoration of the susceptibility of bacteria to antibiotics, the effect revealed in our previous works [5,6]. In contrast, after the application of six antibiotics in vitro, the adhesive and anti-lysozyme activities of seven studied bacteria increased by 35 and 8%, respectively. While in vivo antibiotic application practically did not change these parameters. Obtained results contribute to the perspective of future application of AgNPs for recovering the activity of antibiotics, which nowadays disappear from the market due to fast-growing bacteria drug resistance.

## Figures and Tables

**Table 1:** Comparison of the effects observed after treatment of cow subclinical mastitis with antibiotics and AgNPs in vitro and in vivo.

Parameter	Experiment	Observed Effect		
		After Treatment with AgNPs	After Treatment with Antibiotics	Total Benefit from AgNPs Use Compared with Antibiotic Use
Treatment time, days	In vivo	2.9	7.1	4.2 days (2.5 times faster)
Change of number of isolates, %	In vivo	−64.1	+10.0	74.1
Change of adhesive capacity, %	In vitro	−29.1	+35.2	64.2
	In vivo	−23.1	+1.45	24.5
Change of anti-lysozyme activity, %	In vitro In vivo	−11.3 −22.7	+8.2 −2.2	19.5 20.5

**Table 2:** Examples of the influence of different compounds on bacteria adhesive activity.

Compound	Mechanism	Bacteria	Adhesive Activity Measurement Method	Effect on Adhesion	Experiments	Ref.
AgNPs stabilized by PVP and protein hydrolysate	-	*S. aureus*, *S. epidermidis*, *S. dysgalactiae*, *S. agalactiae*, *S. pyogenes*, *P. aeruginosa*, *E. coli*	Standard method of V.I. Brilis (cow erythrocytes with a concentration of 10^8^ cells/mL)	Inhibition adhesive capacity for 7 bacteria in average: in vitro by 29.1%, in vivo by 23.1%	In vitro In vivo	Present work
AgNPs synthesized with *Rhodomyrtus tomentosa* Leaf extract and pure extract		2 *Staphylococcus* isolates 2 *Staphylococcus aureus* ATCC 29213 and *S. epidermidis* ATCC 35984	Microbial Adhesion to Hydrocarbon (MATH) Test Modified excision-based sampling method	10^6^ CFU/mL decreased to 10^5^–10^3^ CFU/mL	In vitro In vivo (Bovine udder epidermal tissue)	[4]
Coated glass slides by quercetin (a plant pigment/flavonoid) TiO_2_ and WO_3_ nanoparticles	Antiadhesive activity against *B. subtilis biofilm*	*Bacillus subtilis*	Confocal Laser Scanning Microscopy (CLSM)	Anti-adhesive efficacies were 96.71% and 98.97% for the surface coated by TiO_2_ and 79.35 and 87.10% by WO_3_	In vitro	[19]
Seed-derived ethanol extracts (polyphenols and neolignan) from *Persea americana* Mill (avocado)	Modulation of the quorum sensing system by downregulation of the virulence factors such as *mexT* and *lasA* genes	*Pseudomonas aeruginosa*, 64 mg/mL *Staphylococcus aureus*, 64 mg/mL *Escherichia coli*, 512 mg/mL *Staphylococcus pneumoniae*, 128 mg/mL	Standard viable plate count method Inhibition of the bacterial adhesion to A549 lung epithelial cells after 60 min of incubation	10^8^ CFU/mL decreased to 10^3^–10^6^ CFU/mL 10^6^ CFU/mL decreased to 10^1^ CFU/mL 10^16^ CFU/mL decreased to 10^3^ CFU/mL 10^3^ CFU/mL decreased to 10^−1^–10^1^ CFU/mL	In vitro	[20]
Multivalent adhesion molecule coupled to polystyrene microbeads	Blocking pilus assembly	*Pseudomonas aeruginosa*	Adhesion was not measured directly; other parameters were measured	Inhibition or mostly reduced 22 and 30%	In vivo rat model	[21]
Chitosans	Inhibition of the growth and adhesion of human uropathogens on urinary catheters	*Klebsiella pneumoniae* *E. coli*	Colony forming units were enumerated in the plates	Inhibition pH = 5, 10^7^ CFU/mL -> 10^2^–10^3^ CFU/mL pH = 6, 10^7^ CFU/mL -> 10^5^ CFU/mL, 10^6^ CFU/mL -> 10^4^ CFU/mL pH = 5, 10^7^ CFU/mL -> 10^2^–10^4^ CFU/mL pH = 6, 10^7^ CFU/mL -> 10^5^	In vitro	[22]
Salvianolic acid B	Anti-pili of *N. meningitidis*	*Neisseria meningitidis*	A microtiter plate assay	Inhibition of *Meningococcal pili* binding to bovine thyroglobulin (80–93%)	In vitro	[23]
*Phaleria macrocarpa* plant extract	*S. mutans* adhering to the glass surface in the presence of 5 *P. macrocarpa* extracts (6.5 mg/mL)	*Staphylococcus aureus*	Spectrophotometer at 600 nm	Adhesion decreases from 100% down to 10–26%	In vitro	[24]

## Data Availability

The data presented in this study are available on request from the corresponding author.
